# Modeling ChIP Sequencing In Silico with Applications

**DOI:** 10.1371/journal.pcbi.1000158

**Published:** 2008-08-22

**Authors:** Zhengdong D. Zhang, Joel Rozowsky, Michael Snyder, Joseph Chang, Mark Gerstein

**Affiliations:** 1 Department of Molecular Biophysics and Biochemistry, Yale University, New Haven, Connecticut, United States of America; 2 Department of Molecular, Cellular, and Developmental Biology, Yale University, New Haven, Connecticut, United States of America; 3 Department of Statistics, Yale University, New Haven, Connecticut, United States of America; 4 Interdepartmental Program in Computational Biology and Bioinformatics, Yale University, New Haven, Connecticut, United States of America; 5 Department of Computer Science, Yale University, New Haven, Connecticut, United States of America; Stanford University, United States of America

## Abstract

ChIP sequencing (ChIP-seq) is a new method for genomewide mapping of protein binding sites on DNA. It has generated much excitement in functional genomics. To score data and determine adequate sequencing depth, both the genomic background and the binding sites must be properly modeled. To develop a computational foundation to tackle these issues, we first performed a study to characterize the observed statistical nature of this new type of high-throughput data. By linking sequence tags into clusters, we show that there are two components to the distribution of tag counts observed in a number of recent experiments: an initial power-law distribution and a subsequent long right tail. Then we develop in silico ChIP-seq, a computational method to simulate the experimental outcome by placing tags onto the genome according to particular assumed distributions for the actual binding sites and for the background genomic sequence. In contrast to current assumptions, our results show that both the background and the binding sites need to have a markedly nonuniform distribution in order to correctly model the observed ChIP-seq data, with, for instance, the background tag counts modeled by a gamma distribution. On the basis of these results, we extend an existing scoring approach by using a more realistic genomic-background model. This enables us to identify transcription-factor binding sites in ChIP-seq data in a statistically rigorous fashion.

## Author Summary

ChIP-seq is an apt combination of chromosome immunoprecipitation and next-generation sequencing to identify transcription factor binding sites in vivo on the whole-genome scale. Since its advent, this new method has generated much excitement in the field of functional genomics. Proper computational modeling of the ChIP-seq process is needed for both data scoring and determination of adequate sequencing depth, as it provides the computational foundation for analyzing ChIP-seq data. In our study, we show the characteristics of ChIP-seq data and present in silico ChIP sequencing, a computational method to simulate the experimental outcome. On the basis of our data characterization, we observed transcription factor binding sites with excessive enrichment of sequence tags. Our simulation results reveal that both the genomic background and the binding sites are not uniform. On the basis of our simulation results, we propose a statistical procedure using the more realistic genomic background model to identify binding sites in ChIP-seq data.

## Introduction

Gene expression is carefully regulated in all living cells. Only a fraction of the genes in a genome are expressed to various degrees under a given condition or in a particular cell type. The main control of such regulation occurs at the transcription level: the RNA polymerases transcribe genes following binding of trans-acting transcription factors to cis-acting regulatory DNA sequences within genes or in their vicinities. To determine the biological functions of transcription factors, it is imperative to identify their binding sites and target genes in the genome.

Currently the most commonly used high-throughput method for identifying transcription factor binding sites (TFBSs) is chromatin immunoprecipitation followed by microarray hybridization (ChIP-chip) [1]–[3]. In this method, the transcription factors are cross-linked to DNA under the test condition. After the genomic DNA is isolated and fragmented by sonication, an antibody specific to the transcription factor of interest is used to isolate the transcription factor and the DNA fragments which it binds. Following chromatin immunoprecipitation, the protein–DNA crosslink is reversed and the DNA fragments are hybridized to a tiling microarray. After the signal quantification, the DNA fragments enriched by the binding of the transcription factor are identified—in terms of both genomic sequence and location—by the oligonucleotide tiles that give significantly high relative signals on the microarray [4].

Instead of using microarrays to identify the sequences of the immunoprecipitated DNA fragments, new methods have recently been developed to take advantage of the fast-maturing next-generation massively parallel sequencing technologies. In one such method, ChIP-PET [5], paired-end ditags (PETs) derived from both ends of the immunoprecipitated DNA fragments are sequenced and mapped to the genome. In a newer method, ChIP-seq [6],[7], immunoprecipitated DNA fragments are directly sequenced at one end for ∼30 bp, and the short sequence reads are then mapped to the reference genome. The apt combination of ChIP and next-generation sequencing technology has generated much excitement in the field of functional genomics. Comparing with ChIP-chip, whose usability for large mammalian genomes is limited by serious cross-hybridization at high genomic resolution, these sequencing-based methods offer not only direct whole-genome coverage but also low analytical complexity, high signal-to-noise ratio, and sensitivity that increases with sequencing depth. The current trend in high-throughput molecular biology laboratories is to migrate from ChIP-chip to ChIP sequencing to identify transcription factor binding sites in vivo.

Proper computational modeling of ChIP-seq process is needed for both data scoring and determination of adequate sequencing depth, as it provides the computational foundation for analyzing ChIP-seq data. Here we show the characteristics of ChIP-seq data and present in silico ChIP sequencing, a computational method to simulate the experimental outcome. Our simulation results reveal that both the genomic background and the binding sites are not uniform. Such nonuniformity in the background will have important implications in ChIP-seq data analysis and binding sites identification.

## Model

### Characterization of ChIP-seq Data

ChIP-seq data are generated in a straight-forward manner, by high-throughput sequencing and subsequent sequence alignment. Because Illumina/Solexa 1G Genome Analyzer generates a very large number of short sequence reads, ChIP sequencing is currently done mainly with this sequencing platform. This could change in the future, however, as other high-throughput sequencing technologies may become better suited. Here we briefly describe the procedure of ChIP sequencing with the Solexa platform. The immunoprecipitated DNA fragments are sequenced from one end for approximately 30 bp. These short sequence reads are aligned to the human reference genome, and only uniquely mapped reads (typically 60–80% of all sequence reads) are retained for the downstream analysis. Based on size selection after gel electrophoresis prior to sequencing, the retained reads are elongated into longer tags by directional extension to the mean length of the size selected DNA fragments and then transformed into profiles of the number of overlapped DNA fragments at each nucleotide in the reference genome [6].

**Figure 1:**
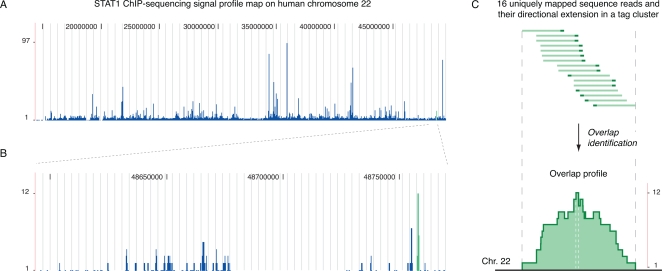
The genomic profile of ChIP-seq data. (A) The signal profile map of STAT1 ChIP-seq data on human chromosome 22. (B) The same signal profile map in a small genomic region on human chromosome 22. (C) The sequence tags and the overlap profile of a tag cluster. This cluster, simplified as green lines in (A) and (B), is defined by 16 sequence tags, each of which is a uniquely mapped sequence read (dark green) plus its directional extension (light green). The outer and the inner forms of this cluster, bound by the two gray and the two white dashed lines respectively, have corresponding tag counts 16 and 12.

For our analysis, we link overlapping tags into tag clusters (Figure 1), each of which is characterized by *y*, the number of tags it contains, and indexed by *a* and *b*, its start and end genomic locations. Thus, by definition a tag cluster is a genomic site continuously covered by one or more sequence tags can be characterized in two different ways. One type of characterization is to set *a* and *b* to the boundaries of the cluster and *y* to the number of all tags in it, while the other is to identify the peak of the overlap in the cluster first and then to set *a* and *b* to the start and the end positions of the peak and *y* to the height of the cluster. We term tag clusters characterized by these two methods as ‘outer clusters’ and ‘inner clusters’ respectively and use ‘outer clusters’ in our analysis. Suppose there are *M* tag clusters, ChIP-seq data after preprocessing are defined by the matrix **T**, whose row *m*, (*a_m_*, *b_m_*, *y_m_*), characterizes tag cluster *m* (*m* = 1,…, *M*). The main goal of our ChIP-seq data analysis is to identify tag clusters that are transcription factor binding sites by determining a threshold on the tag count to separate the DNA-binding signals from the background noise.

### Determination of the Threshold for TFBS Identification

To identify transcription factor binding sites in ChIP-seq data, we assess the statistical significance of each tag cluster found in the actual data by assigning it a *P*-value as the result of the test of the null hypothesis that its tag count is generated by a null distribution, which is the distribution of the tag count on the genomic background alone. This null distribution is generated by placement of sequence reads onto the genomic background in the absence of binding sites. It is critical to simulate the correct background, as the null distribution generated from it is used to assign *P*-values to all actual tag clusters.

The simulation starts with the removal of sequence gaps and repeats from the genomic region—the entire genome or a part of it—under consideration. It is followed by the random placement of *n* sequence tags, corresponding to the same number of uniquely-mapped sequence reads from the experiment, onto the genomic background, whose distribution of the sampling weight on the nucleotide level could be either uniform or non-uniform. After the tag placement, suppose that *N* tag clusters are identified in the simulated data and the largest one contains *C* tags, thus the null distribution of the cluster tag count is given by the number of tag clusters on each tag count level, 1, 2, …, *C*: 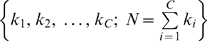.

Given this null distribution, for tag cluster *m* (*m* = 1, 2, …, *M*) identified in the experimental data we calculate its associated *P*-value, *P_m_*, for the test of the null hypothesis that it is part of the background as
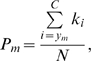
in which *y_m_* is the tag count of tag cluster *m* from the experimental data and *k_c_* is the number of tag clusters on tag count level *c* in the simulated data. In essence this is a permutation test and *P_m_* can be calculated to arbitrary accuracy as the number of simulation increases. To control the type I error in this set of *M* hypothesis tests, we first adjust the *P*-values so that they directly reflect the controlled false discovery rates [8], and then choose the lowest tag count that gives a low FDR (e.g., less then 0.05) as the threshold. Tag clusters with at least this tag count are identified as the binding sites.

### Simulation of the ChIP-seq Process

**Figure 2:**
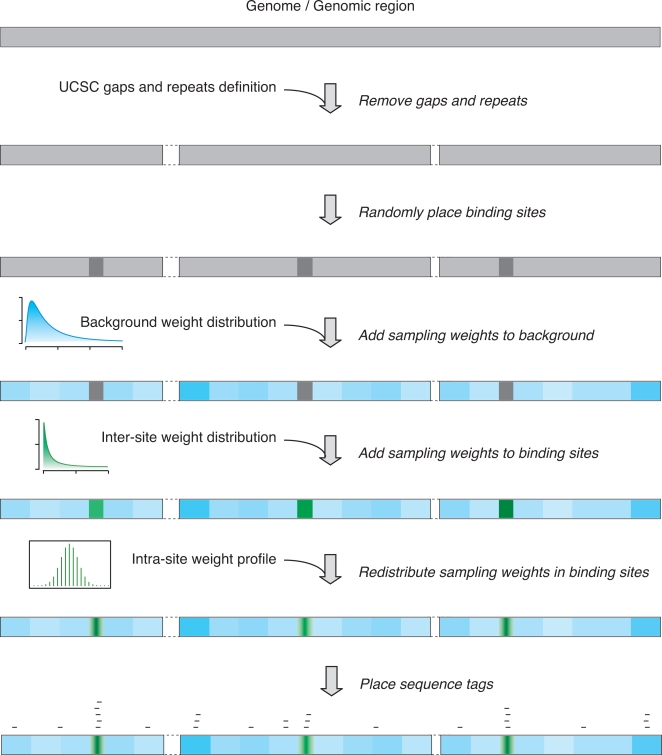
In silico ChIP sequencing. The white segments with dashed borders represent the removed sequence gaps and repeats. The 1-Kb background blocks are rendered by the blue segments with a darker blue for a higher sampling weight. The 500-bp binding sites are represented by the dark gray boxes before sampling weight assignment and green boxes afterwards with a darker green for a higher sampling weight. Notice if a background block has a sampling weight high enough, it can “attract” a similar number of tags as a binding site can. See the main text for a detailed description of the procedure.

For our simulation of ChIP sequencing (Figure 2), we use the lengths of human chromosomes as specified in the NCBI v36/hg18 human genome assembly. We first remove all sequence gaps as defined in the UCSC genome browser annotation database. Because only uniquely mapped sequence reads are used in ChIP-seq data analysis, we also remove positions covered by repetitive sequences identified by RepeatMaster, and then randomly place without overlap a chosen number of transcription factor binding sites, each of which was assumed 500 bp long, onto the genome. After the placement of binding sites, the genome (excluding removed sequence gaps and repeats) is effectively partitioned into the floating fixed foreground (binding sites) and the background.

The process of the chromosomal immunoprecipitation and the subsequent unique mapping and extension of sequence reads can be simulated by randomly placing uniquely mapped sequence tags onto the chromosome, according to certain sampling weight at each nucleotide position. Such weights are generated first for the background nucleotide positions and then for those in the binding sites. For a uniform background, every nucleotide position in the background is given one as its sampling weight. For a varying background, if we assign each nucleotide position a different weight, given the large size of the human genome it becomes computationally prohibitive to sample the background many times as the simulation requires. Instead, we partition the background into adjacent blocks of nucleotide positions. After testing different block sizes ranging from 500 bp to 5 kb, we find they all give practically identical simulation results. In the end, we choose 1 kb as the block size. Every adjacent 1-kb block in the background is given a random weight drawn from a pre-specified underlying distribution and all nucleotide positions in a block are assigned the same weight. For the background variation, we assume that most of the background has a low sampling weight as most of the background is not enriched in the immunoprecipitation (the working principle of ChIP) but a few places of it have relatively high weights, comparable to some binding sites. Based on this assumption, we use a gamma distribution, *Gamma*(*s*,*c*), which skews to the right, for the distribution of sampling weight on the background.

To specify the sampling weights in the binding sites, we first calculate *w̅* *_b_*, the average sampling weight at each nucleotide position in the background, and multiply it by the enrichment coefficient *t*, to obtain *w̅* *_f_* = *t*⋅*w̅* *_b_*, the average sampling weight at each nucleotide position in the binding sites. The ChIP enrichment at different binding sites is, however, different and can be estimated by the fold increase of tags placed in the foreground over those placed in the background in the simulation. Given *w̅* *_f_* and the number of nucleotides in the binding sites, we calculate *W_f_*, the total amount of sampling weight in the binding sites, and then distribute it to each binding site either evenly or varyingly according to a certain distribution. For the intersite variation, we use a power-law distribution generated by a “preferential attachment” procedure. If a tag is placed in a binding site, the current sampling weight of this site, *w_k_*, is updated by a linear function as *w_k_* = *w*+*r*·*k*·*w*, in which *w* is its initial sampling weight, *k* is the number of tags placed at this site, and *r* is the weight increase coefficient. For each binding site, we also distribute the amount of its sampling weight to each nucleotide position according to a symmetric binomial or an equilateral triangular profile. We test various combinations of values for *s*, *c*, and *r*, the two free parameters in our simulation method, and find *s* = 1, *c* = 20, and *r* = 1.5 produce simulated data that give overall best fit to the actual data.

### Implementation

We implemented our ChIP-seq simulation method in R and wrote several auxiliary programs for text processing in Perl. The whole software package with source code and documentation is available for download at http://www.gersteinlab.org/proj/chip-seq-simu.

## Results

### The Observed Tag Count Has a Power-Law Distribution and a Significant Right Tail

For our analysis and simulation of ChIP-seq data, we used the dataset generated from STAT1 DNA binding under IFN-γ stimulation by Robertson et al. [6]. Of the initial 2,915,382 sequence reads obtained in their experiment, 2,025,931 (69.5%) could be uniquely mapped to the unmasked NCBI v36/hg18 human reference genome. After the genomic mapping, we extended the length of mapped sequence reads from 27 to 174 bp, the estimated average length of the size selected DNA fragments [6], and identified 1,264,752 STAT1 tag clusters on the whole genome level.

**Figure 3:**
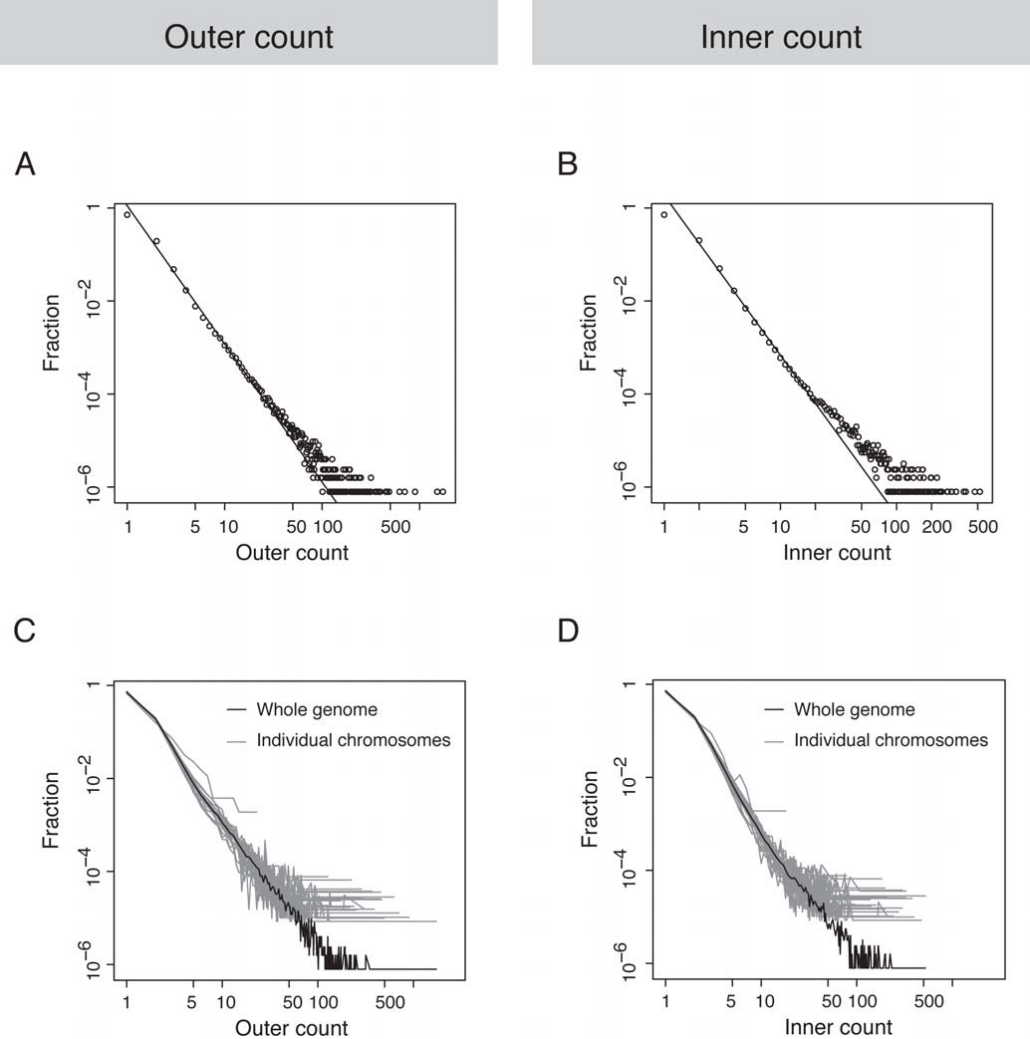
Tag counts from the STAT1 ChIP-seq data. (A) The genome-wide outer counts and their frequencies. The black line is the linear regression on the log-log scale from outer count 2 to 100. (B) The genome-wide inner counts and their frequencies. The black line is the linear regression on the log-log scale also from inner count 2 to 100. (C) The outer counts and their fractions on the whole genome and each individual chromosome. (D) The inner counts and their fractions on the whole genome and each individual chromosome.

While the majority (1,149,405, >90%) of these tag clusters comprise only one or two tags, a relatively small number (661) of them contain large numbers of tags (50 and more, the outer-overlapping count) and consequently show high stacking peaks (the inner-overlapping count) in their profiles (Figure 3A). For example, the most prominent STAT1 tag cluster appears immediately upstream to the centromere of chromosome 1. With a peak height of 472 tags, it comprises 1,733 tags in its ∼1.6 Kb genomic footprint. Indeed, a closer examination revealed that the tag count *c* follows a power-law distribution:
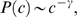
where the degree exponent *γ* = 2.97 (*R* ^2^ = 0.9955, *P*-value<2×10^−16^) for the outer count and 3.44 (*R* ^2^ = 0.9976, *P*-value<2×10^−16^) for the inner count, respectively (Figure 3A and 3B).

We also examined tag counts on individual human chromosomes separately to check for possible discrepancies in their distributions on different chromosomes. The plots in Figure 3C and 3D show that over all the tag count on individual chromosomes and on the genome as a whole follows the same power-law distribution, and there is considerable variation among different chromosomes in the distribution at high counts.

### Both the Genomic Background and the Binding Sites Are Not Uniform

**Figure 4:**
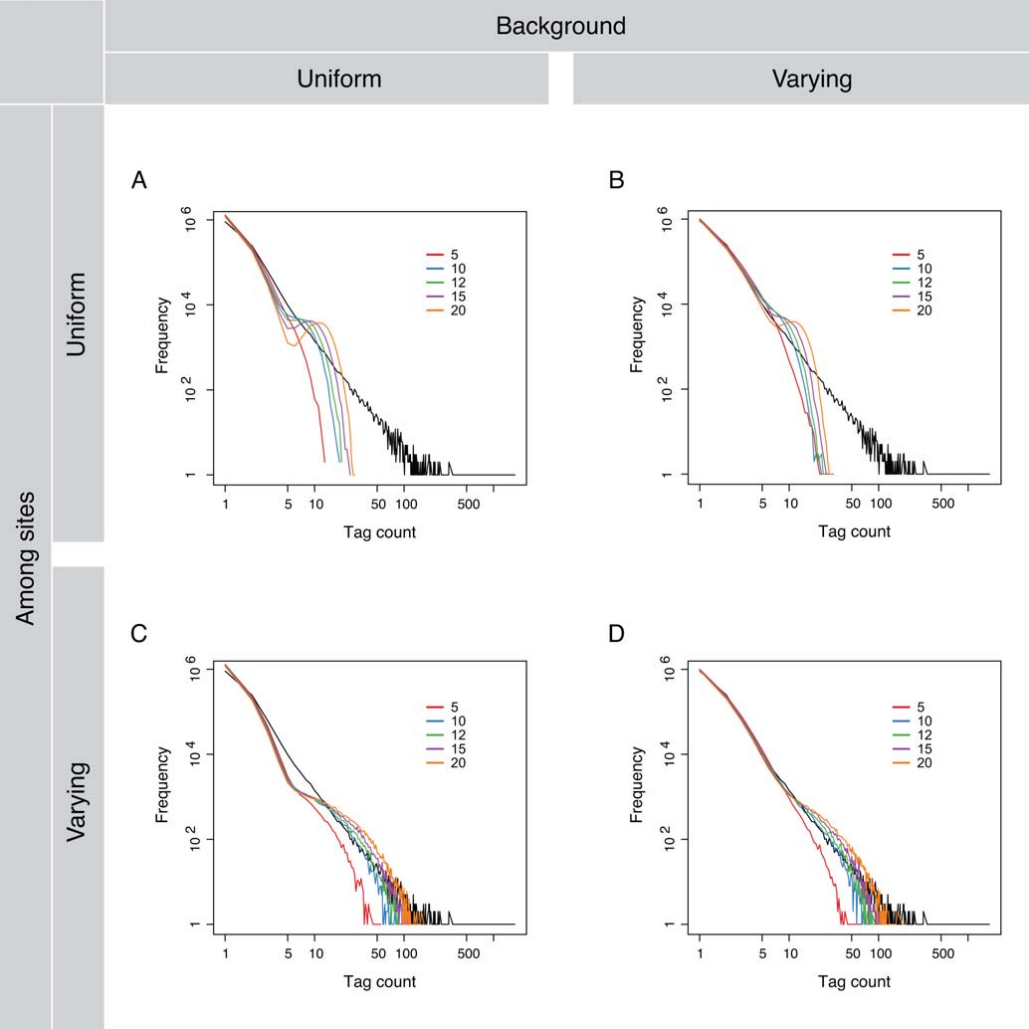
ChIP-seq simulation with different background and binding-site models. (A) Uniform background and uniform binding sites. (B) Varying background and uniform binding sites. (C) Uniform background and varying binding sites. (D) Varying background and varying binding sites. The outer counts are used for all for plots. In each plot the actual tag count distribution is plotted as the black line and five simulated distribution with the enrichment coefficient *t* = 5, 10, 12, 15, and 20 are depicted by the colored lines.

In our simulation of the ChIP-seq process, we use either uniform or varying sampling weights on the genomic background and among the binding sites for the tag placement. The four simulated datasets generated from the resultant combinations of the background and the inter-site distributions fit the actual data in very distinct ways (Figure 4). The goodness of fit is assessed by the fit of the simulated distribution to the actual one in the range of small to high tag counts.

The four combinations of the background and the inter-site distributions can be seen as a gradual increment in the overall simulation complexity: from a simple model that assumes uniformity in both the background and the binding sites to one that assumes variation in either of them and to the most complex one that assumes variation in both of them. The simplest model assumes that the tag placement is identical everywhere on the background and also identical among the binding sites. Data generated from this model give a distribution of tag counts that is a very poor fit to the actual one: not only is there a depletion of tag clusters with small to medium tag counts due to an excess of single tags being placed onto the genome, but also clusters with large tag counts are completely absent (Figure 4A and see the Table S1 for the quantification of the goodness of fit).

The slightly more complicated second model assumes identical binding sites but a varying background for tag placement. The simulated data fit the actual distribution well at small to medium (1 to ∼5) tag counts but there is still a complete absence of clusters with large tag counts (Figure 4B). Contrary to the second model, the third model assumes a uniform background but varying binding sites for tag placement instead. Using this model we see an inversion in the simulation result: tag clusters with small to medium tag counts are depleted in the simulated data while clusters with large tag counts are generated (Figure 4C). Finally, we use a model that assumes variation both in background and among binding sites for tag placement. It generates data that give the best fit to the actual distribution of the tag count in its whole range (Figure 4D).

### The Uniform-Background Model Increases False Identification of Binding Sites

To identify STAT1 binding sites, we can assess the statistical significance of each tag cluster found in the actual data using a null distribution of tag counts derived from a background model. For the initial assessment, we used a simple background model that assumes equal probabilities for random tag placement at every available nucleotide position in the genome and combined 500 independent replicates of background simulation to generate such a null distribution. After assigning *P*-values and adjusting them for multiple testing to control the false discovery rate, we set five and above, which corresponds to an FDR<0.05, as the threshold on the tag count and identify 32,763 STAT1 binding sites.

In light of the simulation results, we can reassess the statistical significance of each tag cluster found in the actual data by using the varying-background model and combining 500 independent replicates of background simulation to generate the null distribution of the tag count. As before, we assign *P*-values to tag clusters found in the actual data by using this null distribution and adjust them for multiple testing to control the false discovery rate. At the same FDR level (<0.05), we set thirteen and above as the threshold on the tag count and identified 5,858 STAT1 binding sites from the initial ∼3-million sequence reads.

Using the full sets of reads, we identified 28,434 and 5,307 STAT1 binding sites with and without IFN-γ stimulation respectively (Table S2). In their study, Robertson et al found 41,582 and 11,004 sites in these two datasets. The reduction in both of our numbers reflects a more stringent threshold for peak calling, which was set by the more realistic varying-background model. Moreover, the proportionally greater decrease in the number of sites without stimulation reflects the limitation of STAT1 as a transcription factor without IFN-γ stimulation. To demonstrate the validity of the threshold change, we performed a STAT1 motif analysis in the peaks that are between the thresholds set by the uniform background and the varying background models. Using Meta-MEME [9] with blocksize = 128,205 characters, background = peaks.bg (nucleotide frequencies estimated from the input peak sequences), and *E*-value<1, we are able to identify significant STAT1 motifs (as defined in TRANSFAC [10] and JASPAR [11]) in 6.1% of those peaks. This result suggests that the threshold increase greatly boosts the specificity at a very small expense of the sensitivity.

**Figure 5:**
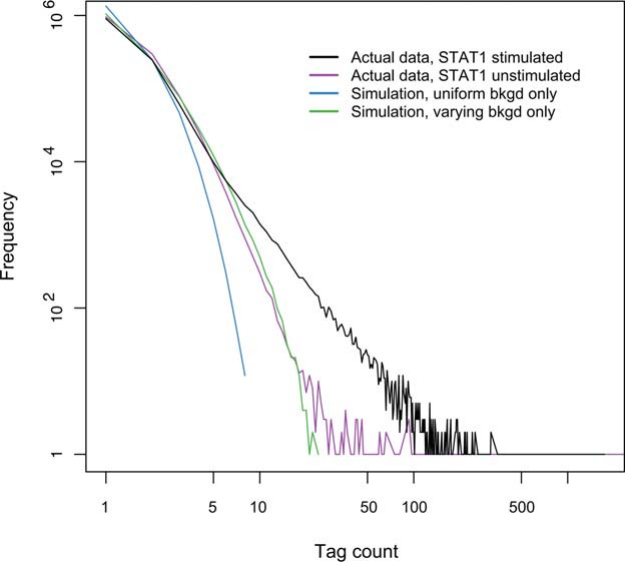
The null and the actual distributions of the tag count. Plotted in blue and green, respectively, two null distributions are generated with the uniform- or the varying-background models. The actual distributions with and without IFN-γ stimulation are depicted by the black and purple lines.

Four distributions of tag counts are plotted in Figure 5: two actual distributions generated by experiments with and without IFN-γ stimulation and two null distributions derived from the uniform- and the varying-background models. Compared with either null, there is a significant increase of the number of tag clusters with high tag counts in the observed stimulated distribution. For example, there are 661 tag clusters with 50 or more tag counts in the actual data but none in the simulated data generated with either background model. While the number of tag clusters strictly decreases monotonously as the tag counts increases in the null distribution, there is a long tail on the right of the actual distribution given by the enrichment of clusters with high tag counts. Moreover, we also observe significant differences between the simulated datasets generated with two background models alone. First, comparing with 903,832 tag singletons in the actual data, there is an enrichment of tag singletons in all simulated background datasets. However, this increase is much more pronounced in the datasets generated with the uniform-background model (∼150%) than with the varying-background model (∼115%). Second, on average there are only three tag clusters with nine or more tag counts in the data simulated with the uniform-background model but over 2,000 with the varying-background model.

To check how closely the varying-background model models the background in the actual experimental data, we compared the distribution generated under this model with the actual one without the IFN-γ stimulation. In response to the stimulation, STAT1 binds to numerous promoter elements to upregulate interferon stimulated genes. Without the stimulation, the role of STAT1 as a transcription factor is limited. Given such a difference in the DNA binding of STAT1 in the presence or absence of IFN-γ, we expect the distribution of tag counts from the experiment without stimulation should be a distribution dominated by a significant background with a small right tail from its limited DNA binding. This is exactly what we see in Figure 5, where the good fit between the distribution simulated under the varying-background model and the actual unstimulated one is striking and shows the validity of the varying-background model. Considering these observations (Figure 5) in the light of the full simulation results presented in the previous subsection (Figure 4), we conclude that as the genomic background is varying it is better captured by the varying model than the uniform one.

## Discussion

### Simulation of ChIP Sequencing

We generate synthetic ChIP-seq datasets under simulation models with various assumptions for the binding sites and the genomic background. By comparing the simulated dataset with the actual one, we assess the goodness of the assumptions made in each simulation and thus can gain insight into the actual ChIP-seq data generating process: the closer the simulated dataset is to the actual one, the closer the assumptions are to the real process.

We use the uniform and the varying models for both the background and the binding sites in our simulation. In Figure 5, marginal comparisons show that the model with a varying (non-uniform) weight distribution for either the background or the binding sites generates substantially better simulated data. When the background and the binding sites are considered together, the simulated datasets generated with various combinations of the background and the binding-site models show striking differences in their quality. The data simulated with the uniform-weight models used for both the background and the binding sites show practically no fitting to the actual data except for the general trend (Figure 5A). When the varying-weight model is used for either the background or the binding sites, there are substantial improvements to the fit in different ranges of the tag count (Figure 5B and 5C). However, when the varying-weight models are used for both the background and the binding sites, not only is the fit the best but also there is a general agreement between the simulated and the actual data (Figure 5D).

These simulation results clearly show that neither the binding sites nor the background is uniformly presented in ChIP-seq data. Due to the inherent random noise in the experiment, binding sites are unlikely to contain the same number of mapped sequence tags. Not all the variance in the number of sequence tags mapped to binding sites could be explained by random noise, which should be counted by the uniform-site model as the simulation itself is intrinsically a stochastic process. Because DNA segments containing the binding sites are enriched by immunoprecipitation, the variance should also reflect the different DNA-binding affinity that a transcription factor has for its binding sites. Such variation could be the result of differences in either the nucleotide sequences of the binding sites [12] or the local chromotin modification status [13].

Perhaps more importantly, our simulation results also reveal that there is a substantial variation in the tag placement on the genomic background. Obviously, such background variation cannot be explained by the uniformity of background currently assumed in ChIP sequencing. Instead, our results suggest a varying background that is mildly fluctuating and contains some “hot” spots with relatively high ChIP enrichment comparable to some binding sites. The presence of such background ‘hot’ spots in the ChIP-seq data may be caused by preferential sequencing particular to the sequencing protocol/platform used in the experiment. Their enrichment through immunoprecipitation is precluded, however, as the background DNA segments are not bound by the transcription factor. Our inference of a varying genomic background not only raises questions about both biology and technology involved in ChIP sequencing but also has important practical implications to the analysis of ChIP-seq data as it provides a better background model (see next subsection for explanation).

**Figure 6:**
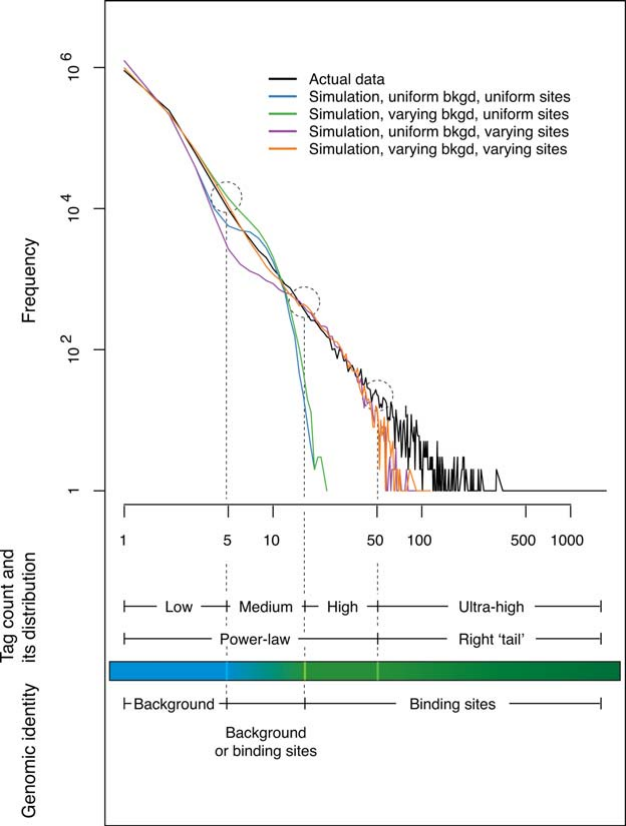
The four segments in the range of the tag count in the actual data. Only the actual tag count distribution and four simulated ones, one (the green line) from each panel of Figure 5 that are generated with the enrichment coefficient *t* = 10 only, are plotted here for clear depiction. Notice the convergence at the start or the end of each of four pairs of simulated distributions generated with the same background or site models. For example, the blue and the green curves differ at the start but converge at the end because they are generated with different background models but the same site model. The range of the tag count in the actual data is divided into four segments based on the divergence and convergence (indicated by dashed circles) of the actual and simulated distributions. These sections correspond to different features of the actual tag count distribution and the genomic identities of the tag clusters.

To examine our simulation results more closely, we plot in Figure 6 the actual tag count distribution and the simulated ones generated under different background and site models with the enrichment coefficient *t* = 10 only (the blue lines in Figure 5A–D) because as seen in Figure 5D at this enrichment level the simulated data give the best fit to the actual ones. Based on the fitting of different simulated distributions to the actual one, the range of the tag count in the actual data can be divided into four sections with low, medium, high, and ultrahigh tag counts respectively.

As marked by the dashed circles and lines in Figure 6, the three section boundaries are defined by the divergence of the simulated distribution based on the varying-background and uniform-site model from the actual distribution (the green and the black lines), the convergence of the simulated distribution based on the uniform-background and varying-site model from the actual distribution (the purple and the black lines), and the divergence of the simulated distribution based on the varying-background and varying-site model from the actual distribution (the orange and the black lines). Based on the models used to generate these simulated distributions, we can also infer the genomic identities of tag clusters found in the actual data. Tag clusters with low and high (including ultrahigh) tag counts are almost certain to be background and binding sites, respectively. Because there is a mixture of signals, the true identities of the clusters with medium tag counts are much less certain, and thus some form of thresholding is necessary. Figure 6 also shows that the part of the tag count that has a power-law distribution is supported by the background or the binding sites or both at low, high, and medium counts respectively. The right tail, diverged from the power-law distribution (Figure 4), occupies the ultra-high count section.

### Methods To Identify Binding Sites in ChIP-seq Data

Reported in two recent studies [6],[7], ChIP sequencing is a newly-developed high-throughput method for genome-wide mapping of in vivo protein–DNA association. In these two studies, two different analytical methods were used to identify transcription factor binding sites. In the first study [7], a list of sites ‘known’ to be bound (the positives) and unbound (the negatives) by the transcription factor being studied is first compiled. Given this ‘gold standard’, the sensitivity and the specificity of the experiment at each threshold on the sequence read per region are then calculated. And finally a threshold is chosen to give both high sensitivity and high specificity. In the second study [6], a background model is first used to simulate the sequence read placement unto the genome in the absence of binding sites. The false discovery rate, defined as the ratio of the number of peaks at and above a peak height threshold in the simulated data to that at and above the same threshold in the actual data, is then calculated at each peak height as the threshold. And finally a threshold on the peak height is chosen to give a stringent FDR. For easy reference in our later discussion, we name the former the “known-sites” method and the latter the “background-simulation” method.

The known-sites method has the advantage in giving the sensitivity and the specificity of a particular ChIP-seq experiment at a chosen threshold. Its applicability is, however, problematic since it requires a “gold standard,” a list of true positives and true negatives. Conceptually, the validity of such a ‘gold standard’ is questionable given the dynamic nature of protein–DNA association—i.e., under different conditions a transcription factor has different DNA-binding profiles. Operationally, this method is also difficult to use. The prerequisite functional “gold standard” is rarely available, let alone a good one. Moreover, the “known” positives are biased towards binding sites with high enrichment of sequence tags, and as the majority of the genome is not bound by a transcription factor ever, it is an open question how many “true negatives” should be included in the calculation. That is, given the huge preponderance of negatives, it is very difficult to build a correctly balanced gold standard, which is essential for training an effective classifier [14].

Instead of using a “gold standard” to identify binding sites in ChIP-seq data, the background-simulation method uses a background model to simulate how sequence reads are distributed in a genome in the absence of binding sites. Since this method does not assume any prior knowledge about the binding sites of the transcription factor under investigation, it avoids major difficulties encountered by the known-sites method. In their study, Robertson et al used a background model that implicitly assumes uniform tag placement everywhere on the background. However, our simulation results show that the data generated by this uniform-background model agree poorly with the actual experimental data. Based on our further analysis, we can generate a better null distribution by using a more realistic, varying-background model that assumes most of the background is not enriched but at a few places it has a high enrichment level on a par with some binding sites.

In our analysis we estimated the background and the foreground together from the ChIP-seq sample data alone. However, if the negative control data from the experiments without immunoprecipitation are available, the estimation of the background becomes simpler as such experiments give a direct empirical estimate of the ChIP-seq background. Because our method can simulate the background alone, the negative control data can thus be easily accommodated. First the control data are used to estimate the parameters of the varying background model. The fitted model is then used to generate the null distribution of the tag count. And finally this null distribution is used to score the ChIP-seq data.

We also make improvement to the usage of the null distribution in the background-simulation method. In the study of Robertson et al, the false discovery rate is defined as the ratio of the number of peaks at and above a threshold in the simulated data to that at and above the same threshold in the actual data. The implicit assumption behind this definition is that the peaks identified in the simulated data are false positives and the number of them is equal to the number of false positives in the actual data. The first half of this assumption is reasonable, but the second half is unwarranted. For direct comparability, the same number of uniquely mapped sequence tags as contained in the actual data is used to simulate the null distribution on the background. Due to the finiteness of this number and the presence of binding sites (the true positives) in the actual data, the number of the peaks identified in the simulated data will be greater than the number of false positives in the actual data at any threshold. This discrepancy is more pronounced at lower thresholds. In fact, at low thresholds there could be more peaks in the simulated data than in the actual data. When this happens, the false discovery rate exceeds one, which is nonsensical. Instead of using the null distribution in such an *ad hoc* manner, we use it to assign each tag cluster found in the actual data a *P*-value to assess its statistical significance. We then adjust the *P*-values of the multiple-hypothesis tests to control the false discovery rate.

## Supporting Information

Table S1 The goodness of fit between the simulated and the actual curves.
(0.03 MB PDF)

Table S2 Publicly available ChIP-sequencing datasets and the number of sites identified using varying-background model.
(0.03 MB PDF)
