# Supplementary material for: Modeling ChIP Sequencing In Silico with Applications
Source: PLoS Comput Biol. 2008 Aug 22;4(8):e1000158. doi: 10.1371/journal.pcbi.1000158 (PMC2507756; doi:10.1371/journal.pcbi.1000158)
Supplement: Table S1 — The goodness of fit between the simulated and the actual curves. (0.03 MB PDF) [file pcbi.1000158.s001.pdf]

Modeling ChIP sequencing *in silico* with applications

Zhengdong D. Zhang, Joel Rozowsky, Michael Snyder, Joseph Chang, Mark Gerstein

Supplementary table 2. Publicly available ChIP-sequencing data sets and the number of sites identified using varying-background model.

Data set	Experimental outcome [1]			Model parameters [2]		n_{site} [3]
	l_r	l_t	n_{umr}	s	c	
Robertson et al						
STAT1 stim	27	174	1,426,879	5	10	28,434
STAT1 unstim	27	174	1,179,413	5	10	5,307
Barski et al						
H3K4me1	24	220	1,066,623	5	15	94,073
H3K9me2	25	220	721,504	10	10	18,639
H3K27me3	25	220	780,377	5	5	29,451

1. l_r, the read length; l_t, the tag length. Both are in base pairs.
   n_{umr}, the number of the uniquely mapped sequence reads.
2. s, the shape, and c, the scale, for the gamma distribution of the fitted varying-background model.
3. n_{site}, the number of sites identified at FDR < 0.05.
