# Supplementary material for: Modeling ChIP Sequencing In Silico with Applications
Source: PLoS Comput Biol. 2008 Aug 22;4(8):e1000158. doi: 10.1371/journal.pcbi.1000158 (PMC2507756; doi:10.1371/journal.pcbi.1000158)
Supplement: Table S2 — Publicly available ChIP-sequencing datasets and the number of sites identified using varying-background model. (0.03 MB PDF) [file pcbi.1000158.s002.pdf]

Modeling ChIP sequencing *in silico* with applications

Zhengdong D. Zhang, Joel Rozowsky, Michael Snyder, Joseph Chang, Mark Gerstein

Supplementary table 1. The goodness of fit between the simulated and the actual curves. [1]

Background: Uniform; Among sites: Uniform

A

Enrichment coef. t	Mean squared error
5	1.586998897
10	0.863164925
12	0.725042018
15	0.754559600
20	0.629474980
Average	0.911848

Background: Varying; Among sites: Uniform

B

Enrichment coef. t	Mean squared error
5	0.990667305
10	0.889633664
12	0.771627405
15	0.679462299
20	0.484540718
Average	0.7631863

Background: Uniform; Among sites: Varying

C

Enrichment coef. t	Mean squared error
5	0.353800760
10	0.092974392
12	0.090004782
15	0.103924481
20	0.131689485
Average	0.1544788

Background: Varying; Among sites: Varying

D

Enrichment coef. t	Mean squared error
5	0.239834829
10	0.004265812
12	0.007199094
15	0.021765468
20	0.042511183
Average	0.06311528

1. See Figure 4 for the corresponding curves in each panel.
